# On the type locality of the Naso Stardrum *Stellifer naso* (Jordan 1889), and comments on morphological change over a century

**DOI:** 10.1111/jfb.70072

**Published:** 2025-05-05

**Authors:** Jonas Andrade‐Santos, Marcelo R. Britto

**Affiliations:** ^1^ Departamento de Vertebrados – Museu Nacional Universidade Federal do Rio de Janeiro, Setor de Ictiologia Rio de Janeiro Brazil; ^2^ Programa de Pós‐Graduação em Ciências Biológicas (Zoologia) – PPGZoo, Museu Nacional, Universidade Federal do Rio de Janeiro Rio de Janeiro Brazil; ^3^ IUCN Species Survival Commission ‐ Croaker and Drum Fishes Red List Authority

**Keywords:** Dam, drum fish, mislocality, Thayer expedition

## Abstract

*Stellifer naso* is known from the State of Bahia (Brazil), but there is a lack of an accurate definition of its type locality. Thayer's expedition (around 1865–1866), led by Louis Agassiz and staff from the Museum of Comparative Zoology (MCZ, Harvard University), intended to explore Brazilian fauna and flora, with most of its material housed in the MCZ and used to describe several species, including *S*. *naso*. Examination of specimens from the original description of that species, housed in the MCZ and the Smithsonian National Museum of Natural History, helped review the accurate point of origin for such specimens. A combination of data from reproductive biology and the history of the region further support the actual type locality of *S*. *naso* as Iguape Bay, with an error radius of 2.5 km. Damming in the Paraguaçu River in the early 1980s might have had an impact on the morphological variation over time recorded to this species.

## 1 INTRODUCTION

Several expeditions made by foreign naturalists have surveyed Brazilian waters since the sixteenth century and material originating from those expeditions was used in several fish descriptions. However, most of these descriptions provided inaccurate locality (Rosa et al., 2023). An effort has been made to increase access to detailed information about those expeditions, either detailing expeditions themselves (e.g. Thayer's expedition; Higuchi, 1996) or cross‐checking type localities with fish species (e.g. Spix and Martius; Koerber, 2021). Thayer's expedition derived from an old desire of Agassiz to study the Brazilian flora and fauna, especially freshwater fishes, and an attempt to refute views on the evolution of species, which was at the time recently published by Darwin (Britski & Figueiredo, 2019; Dick, 1977). Biological material gathered by this expedition, including watercolours and notes, was deposited at the Museum of Comparative Zoology (MCZ, Harvard University). Comprehensive compilations on the routes, locations and watercolour identifications provided a better understanding of specimens collected during Thayer's expedition, mainly fish species (Britski & Figueiredo, 2019; Dick, 1977; Higuchi, 1996). Accordingly, several sciaenid fishes were collected, largely studied by authors such as Eigenmann, Jordan and Steindachner. In a review of Sciaenidae (Jordan & Eigenmann, 1889), Jordan described the Naso Stardrum *Stellifer naso* based on material from Thayer's expedition (MCZ 4583 from which derived the five syntypes catalogued at Smithsonian Fish Collection USNM 130630).

With a surface area of over 50,000 km^2^, the Paraguaçu River is the second largest river restrict to the Brazilian state of Bahia (i.e. its headwaters and river mouth are in the state), and as such it is of great value to local communities as source of water and food resources (Barros et al., 2008; INEMA, 2023). In the sixteenth century, the Portuguese colonization of Brazil led to the development of the village of Vila de Nossa Senhora do Rosário do Porto da Cachoeira, currently known as the municipality of Cachoeira, named after the waterfall (‘cachoeira’ in Portuguese) that used to delimit this village, the furthest navigable point in the lower Paraguaçu River. The damming of the river in the 1980s prevents flooding in Cachoeira and São Felix, changing the livelihood dynamics of both towns and promoting changes in the state of the river by reducing the natural freshwater input to its estuary (Barros et al., 2008; Genz et al., 2008). As a result, the marine inflow into the estuary overtook the freshwater from the river, turning the part near the dam into an estuary and modifying the fish community, as indicated by the record of marine species in this region (Reis‐Filho et al., 2010). Additionally, with the loss of Atlantic rainforest coverage, currently the land use in the surroundings of the river is dominated by urban and agricultural expansion (Ornellas et al., 2022), such as piassava and dendezeiros (palm trees) plantations (Kuhn, 2009). Since 2000, this area around Cachoeira has been under the domain of the RESEX Iguape Bay (Marine extractive reserve; ICMBio, 2000), which might have slowed this agricultural expansion (Ornellas et al., 2022).

Fish shape is directly related to habitat use and it is also prone to phenotypic plasticity when human‐made or natural changes occur in their habitat (Acar & Kaymak, 2023; Gilbert et al., 2020; López‐Fernández et al., 2013). As in the macroevolutionary sense lineages present a shape driven by natural selection pressures, in the microevolutionary sense fish shape also follows river conditions as they (or other habitats) change from fast‐flowing to slow‐water current (Gilbert et al., 2020; López‐Fernández et al., 2013). In this sense, as human impacts on the environment increase, we should expect to find more changes in fish shape on ecological time scales (Carroll et al., 2007). In the Paraguaçu River, the construction of the Pedra do Cavalo dam in the 1980s diminished its total freshwater discharge by almost half and consequently decreased its flow velocity (Lima & Lessa, 2002). Hence, before the completion of the dam the river channel was mainly freshwater, with estuarine condition only in the Iguape Bay (Lima & Lessa, 2002).

Although there is information on the type locality of *Stellifer naso*, this is often reported as ‘Cachiura’ (Jordan & Eigenmann, 1889: 395; Higuchi, 1996: THAYER015 station, spelled as ‘Cachoeira’, ‘Caxoeira’) in an inaccurate way. Although such misspellings do not require a thorough revision of the type locality, it is currently associated with a high degree of error in its geographical positioning. Consequently, this may mask the discrepancy between the locality suggested by the biology of the species and that suggested by the museum records. Additionally, the environment has changed since Thayer's expedition. This alteration reduced by almost half the natural flow in this river (Lima & Lessa, 2002), and such an alteration might have driven morphological differences between populations across time.

In this scenario, the purpose of this paper was to generate an accurate geographical position for the type locality of *S*. *naso* through a review of information on this region compared with historical changes and the biological aspects of this species. We also comment on morphological changes likely driven by damming in the lower Paraguaçu River.

## 2 MATERIALS AND METHODS

### 2.1 Data curation

Data on the type locality were based on literature records of *Stellifer naso* (Jordan, 1889), including those of Jordan and Eigenmann (1889), Schultz (1945), Chao (1978), and Casatti and Menezes (2003). A survey on fish collections and their catalogues (MCZ, Harvard University; National Museum of Natural History [USNM], Smithsonian Institute) provided comparative information. Additional material from the Paraguaçu river basin was examined at the Museu de Zoologia da Universidade Estadual de Feira de Santana (MZFS). In parentheses, the first number refers to amount of specimens analyzed. Material examined (all from Brazil): MCZ 4583 (3, 64.6–75.6 mm SL) Cachoeira, Bahia, November 1865; MCZ 10808 (1, 75.8 mm SL) Cachoeira, Bahia, November 1865; MZFS 7013 (3, 94.1–116.0 mm SL) Paraguaçu River, Bahia, July 2005; MZFS 9719 (13, 65.0–98.0 mm SL) Paraguaçu River, Bahia, July 2007; MZFS 9720 (4, 84.4–104.0 mm SL) Paraguaçu River, near Coqueiros–Maragogipe, Bahia, July 2007.

### 2.2 Statistical analysis

We used the following six linear measurements to describe the morphology of fishes: (1) body depth (distance from dorsal‐fin origin to ventral profile, as a ratio of standard length [SL]); (2) caudal peduncle depth (ratio of caudal peduncle depth in its length); (3) eye diameter (horizontal eye diameter expressed as ratio of head length); (4) head elongation (ratio of predorsal length in the head length); (5) second dorsal‐fin spine (ratio of second spine in the third spine); and (6) third dorsal‐fin spine (ratio of third spine in the dorsal‐fin base). We then removed allometry from the data using the package ‘GroupStruct’. We performed principal component analysis (PCA) to verify data dispersion among the groups, using all these measurements except the head elongation. Preliminary analyses indicated high collinearity between some variables (none of the above), which were removed from our dataset. We also tentatively included ‘year’ as a variable, but it showed a signal like other variables and was a categorical rather than continuous state. We also followed the proposal of Andrade‐Santos et al. (2024) for a body depth proxy, and the results were similar to the regular body depth measurement. Thus, based on previous studies in Stelliferinae (e.g. Andrade‐Santos et al., 2024; Chao et al., 2021) and these preliminary analyses, we believe that our dataset contains variables that explain well the diagnosis and variation of this species in the Paraguaçu River. We also performed box plots (overlaid with violin plots) to further investigate morphological changes throughout time based on the data from body depth and head elongation. All analyses were performed in the R platform (R Core Team, 2023). The PCA, multivariate analysis of variance (MANOVA), and box plots were performed using the packages ‘FactoMineR’, ‘factoextra’, ‘ggplot2’, ‘REdaS’, and ‘vegan’. The map was created using the Free and Open Source QGIS.

## 3 RESULTS

### 3.1 Type‐locality spelling correction

**FIGURE 1:**
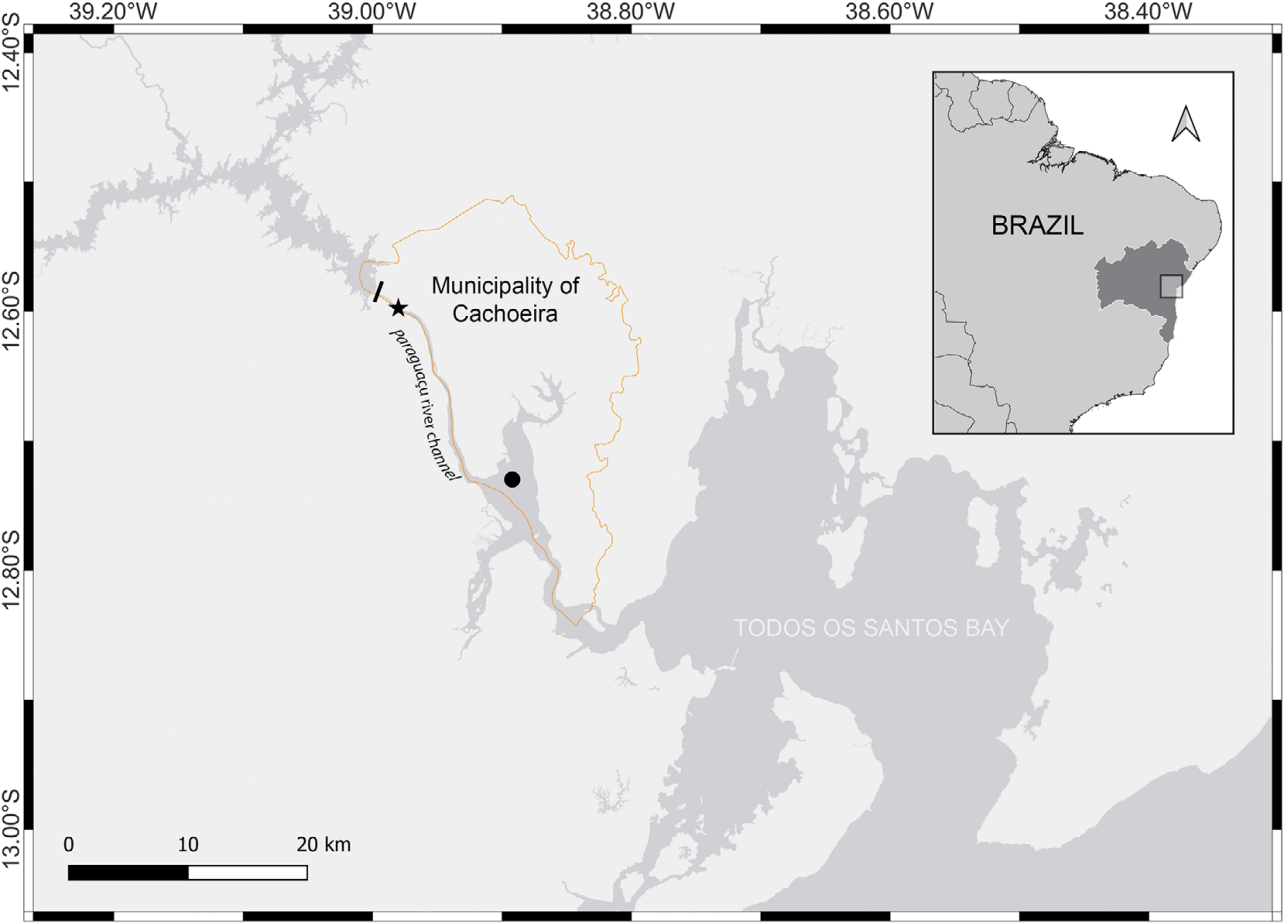
Map of the type‐locality of *Stellifer naso* (Jordan 1889) in the Paraguaçu River and Iguape Bay, which flows into Todos os Santos Bay in the lower‐right corner. The area of the municipality of Cachoeira is represented by the orange line. Black‐filled icons represent the Pedra do Cavalo dam (rectangle), the type locality from previous records (star) and the type locality suggested in the present study (circle). The state of Bahia is indicated by darker grey shading.

*Stellifer naso* (Jordan 1889) is known from its syntypes (USNM 130630) with their locality listed as Cachiura, Bahia, Brazil (Figure 1). The syntypes originated from the lot MCZ 4583, thus herein they are all considered a single population. The misspelling ‘Cachiura’ can be found associated with *S*. *naso* until the mid‐20th century, being the revision of specimens from Jordan's description made by Schultz (1945), the last known use of this misspelling of the type‐locality of *S*. *naso*. In the same work, Schultz (1945) transferred *S*. *naso* to the genus ‘*Ophioscion*’ (nowadays a junior synonym of *Stellifer*; Chao et al., 2021). An alternate misspelling (‘Caxoeira’) and the correct spelling (‘Cachoeira’) are recorded as the locality of the THAYER015 station (Higuchi, 1996). Chao (1978) designated this species to the genus *Stellifer* and referred to ‘Cachoeira’ as the type locality of *S*. *naso*, which was subsequently followed by Casatti and Menezes (2003) in their catalogue of marine fish species in Brazil. After this, the only citation to the misspelling is found in Parenti (2020), which followed Eschmeyer's Catalogue of Fishes (Fricke et al., 2024; cited as 2019 in the original work).

**FIGURE 2:**
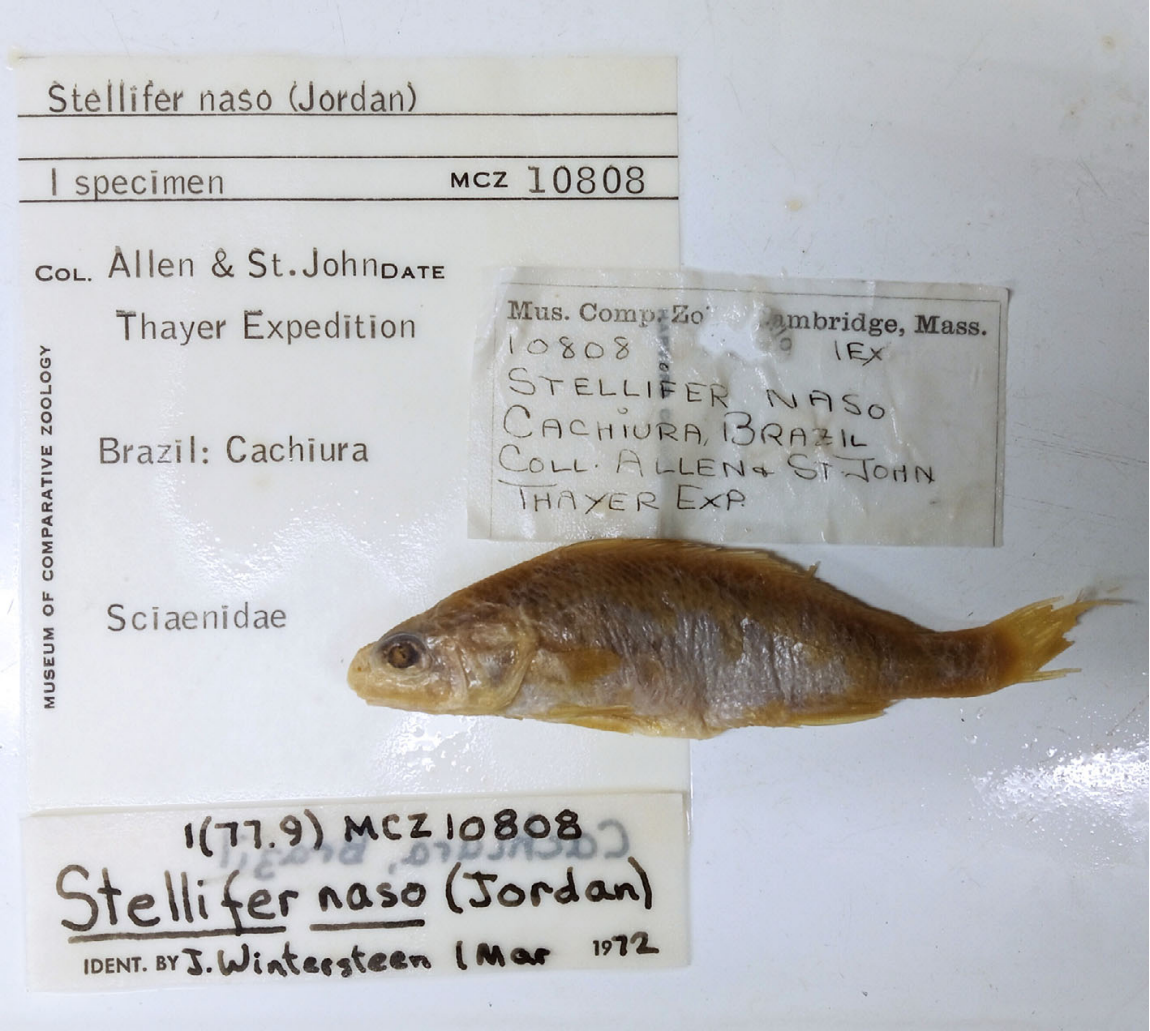
Specimen from the Museum of Comparative Zoology (MCZ 10808) with labels included in the jar.

Records of *S*. *naso* (MCZ 4583, MCZ 10808)^1^ indicate that these specimens were caught in 1865 during one of Thayer's expeditions, at the station THAYER 015. According to Higuchi (1996), this station refers to Cachoeira as its point of origin and Allen (Joel Asaph Allen) as the sole collector. However, accordingly to the MCZBase, which was retrieved from GBIF.org (2024), Agassiz and Bourget were the collectors of MCZ 4583. In addition, handwritten labels (and MCZ's catalogue) in the MCZ 10808 jar include St. John (Orestes St. John) as an adjoined collector with Allen. Because St. John and Allen split ways in Januária (State of Minas Gerais), it is most likely that Allen was the sole collector of *S*. *naso* as his health condition led him to be alone in Bahia before returning to the United States (Britski & Figueiredo, 2019; Dick, 1977). Despite this inconsistency, all information points to municipality of Cachoeira (State of Bahia), Brazil as the type locality of *S*. *naso* (Figure 2). The literature records agree that Allen took a route from the municipality of Jacobina (north‐central region of the state of Bahia) to Cachoeira, passing through municipality of Feira de Santana. Then, he must have gone east through the Paraguaçu channel, collecting *S*. *naso* type‐series on the way to municipality of Salvador (current Bahia state capital), called ‘Bahia’ by Dick (1977).

### 3.2 Type‐locality positioning

**FIGURE 3:**
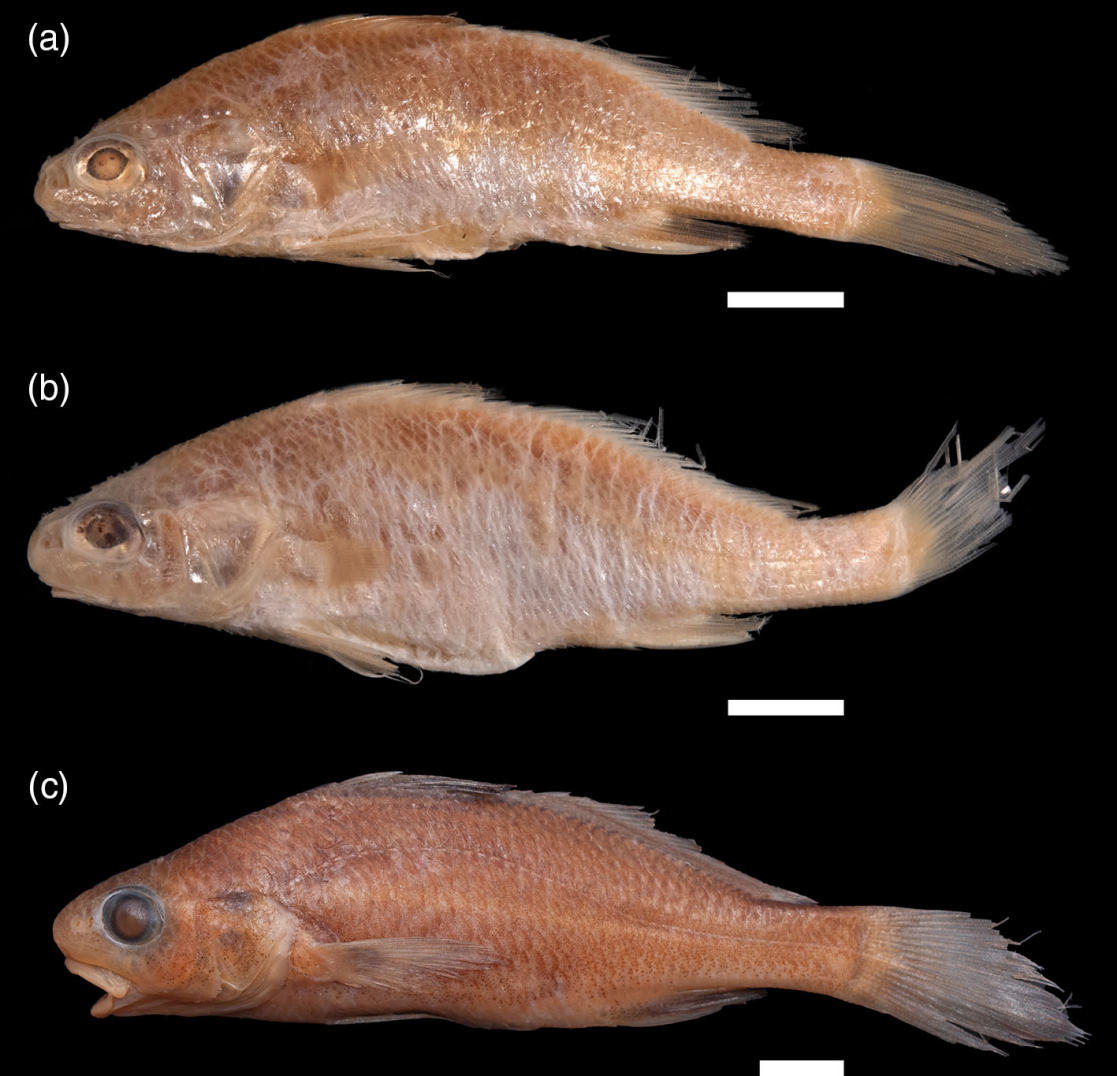
*Stellifer naso* (Jordan 1889) topotype collected with the type series and recently collected specimens: (a) Museum of Comparative Zoology (MCZ) 4583, (b) MCZ 10808 and (c) Museu de Zoologia da Universidade Estadual de Feira de Santana 7013. All specimens were collected in the lower Paraguaçu River channel. Scale bar: 10 mm.

Concerning specific coordinates, the MCZ Ichthyology database indicates 12°35′S, 38°59′W as coordinates to the lots MCZ 4583 and MCZ 10808, with an error radius of 115 km. As locality remarks state, those coordinates are estimated and should probably refer to brackish water. Herein, the accurate type locality is most likely a place inside Iguape Bay (12°43′S, 38°53′W), still inside the area of municipality of Cachoeira, and has an error radius of only 2.5 km. The specimens collected during Thayer's expedition resemble specimens caught more recently between Coqueiros' locality and Iguape Bay (Figure 3). Although the original description is very limited, examination of type specimens allowed identification of this species.

### 3.3 Morphological variation

**FIGURE 4:**
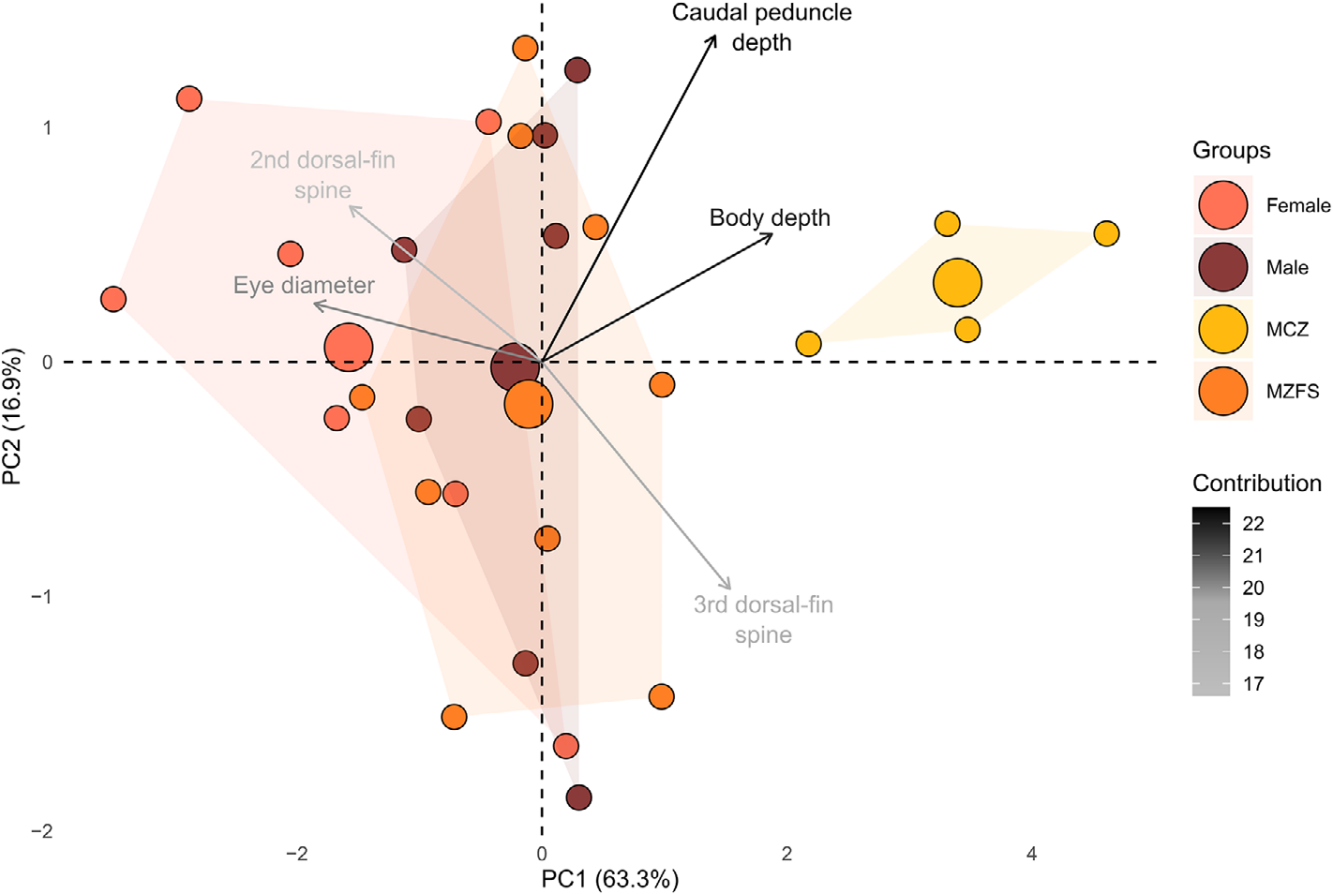
Principal component analysis for morphological data using specimens of *Stellifer naso* (Jordan 1889): male and female (*n* = 7 for each), Museum of Comparative Zoology (MCZ) (*n* = 4) and Museu de Zoologia da Universidade Estadual de Feira de Santana (MZFS, *n* = 9). Sex groups delimited from specimens of MZFS 7013, 9719, 9720; MCZ: 4583, 10,808; MZFS: 7013, 9719.

**FIGURE 5:**
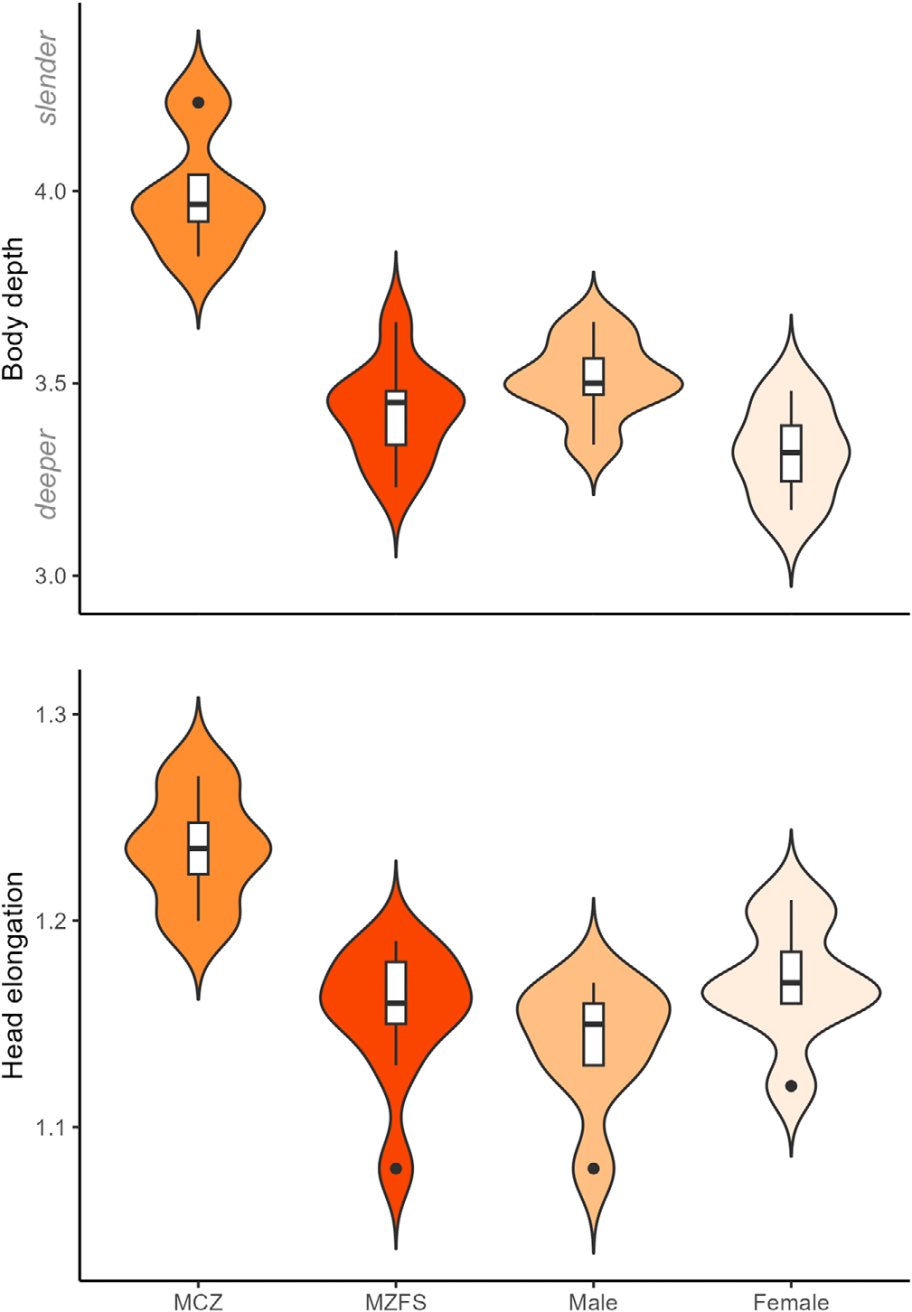
Box plots (with violin plot overlaid) based on head elongation and body depth variation between material from the Museum of Comparative Zoology (MCZ) (specimens from Thayer's; pre‐dam) and Museu de Zoologia da Universidade Estadual de Feira de Santana (MZFS) (specimens from the 2000s; post‐dam). All specimens from the Paraguaçu River (*n* = 27, MCZ 4, MZFS 9, male 7, female 7) were classified by sex to account for sexual dimorphism.

Analyses of the morphology of groups pre‐dam (MCZ 4583, 10,808) and post‐dam (MZFS 7013, 9719, 9720), show a difference between the groups' centroid (Figure 4). These groups are significantly different (MANOVA: *F* = 6.34, *p* < 0.001). The distinction between them can be explained by variations in body and caudal peduncle depth (Table 1). As most of the specimens used by Jordan were females, we categorized the data by sex to rule out that such variation would be from sexual dimorphism rather than differences over time (Figure 4). Between sexes, females tend to have deeper bodies than males, as is usually the case in *Stellifer* (see the exception in Andrade‐Santos et al., 2024). However, this distinction is much lower than those recorded between pre‐ and post‐dam groups (MANOVA: *F* = 8.99, *p* < 0.001; Figure 5). Post‐dam specimens have deeper bodies and less elongated heads in comparison with pre‐dam specimens (Figure 5). Unfortunately, we did not have access to material between 1865 and 1980 to better evaluate this morphological change and the species' description does not cover the morphological variation on the species, a topic that it is part of an ongoing taxonomic revision on the Stelliferinae subfamily.

**TABLE 1:** Principal component (PC) analysis loadings from the first three components for morphological data of *Stellifer naso* (Jordan 1889).

	PC1	PC2	PC3
Body depth	0.96	0.28	0.09
Second spine of dorsal fin	−0.75	0.32	0.57
Third spine of dorsal fin	0.78	−0.49	0.39
Caudal peduncle	0.71	0.70	−0.03
Eye diameter	−0.98	0.13	−0.12

## 4 DISCUSSION

The municipality of Cachoeira (state of Bahia) has a rather large area (about 395 km^2^) in this region (IBGE, 2022) because its territory encompasses the area close to the Pedra do Cavalo dam and the Iguape Bay, on the left margin of the Paraguaçu river (Figure 1). Thus, herein the type locality is likely still in the area of Cachoeira. However, it needed an additional data, that is, Iguape Bay (Cachoeira, State of Bahia). This resulted in a more accurate type locality definition by reducing the error radius from 115 to 2.5 km.

At the time of Thayer's expeditions, before the construction of the Pedra do Cavalo dam, the total freshwater discharge from the Paraguaçu River was about 42.7% higher (mean of 107.7 m^3^/s) than after the dam's final construction in the 1980s (Lima & Lessa, 2002). As stated in MCZbase locality remarks, which can be retrieved at GBIF.org (2024), the specific locality is probably in brackish water. Before the dam was finally built, brackish waters did not reach the Paraguaçu river channel, being recorded only inside Iguape Bay, as the freshwater discharge kept lower salinities (<5) inside the Paraguaçu river channel and brackish waters from Iguape Bay downstream to Todos os Santos Bay (Lima & Lessa, 2002). Nowadays, estuarine/brackish conditions are also present on the river channel and one of the visual differences in this area is the replacement of the Atlantic rainforest by mangrove vegetation, which grows near to the dam, as the inflow of marine waters outweighs the low freshwater discharge (Lima & Lessa, 2002; Ornellas et al., 2022). *Stellifer naso* reproductive biology shows that juveniles (<95 mm total length) seem to prefer less saline areas (7.3–18.5) to grow and the spawning area for adults is in saltier zones (~27) (Camargo & Isaac, 1998, 2005). Following Camargo and Isaac (2005), most of the specimens examined from the original description should be categorized as juveniles. The association between this biological aspect and the historical salinity could place older records of *S*. *naso* somewhere inside Iguape Bay, where they once reached the values of salinity reached by *S. naso* according to Lima and Lessa (2002). Currently, those values are recorded between Iguape Bay and median sites inside the Paraguaçu river channel (Reis‐Filho et al., 2010).

Comparing the morphology between specimens caught in the early 2000s with the ones caught during Thayer's expedition demonstrate that, unexpectedly, they do not overlap in their morphospace and they are statistically a distinct group. This could suggest a historical change in fish shape, as evidenced by the variation in the body and caudal‐peduncle depth. Dam‐driven changes in the riverine state have been suggested as a source of body shape change in fishes (e.g. in body depth), as a slow water current condition (post‐dam) could promote changes towards slender or deeper bodies based on approaches using several river systems and single species (Cureton & Broughton, 2014; Franssen, 2011), a single river system and several species (Geladi et al., 2019; Gilbert et al., 2020). As damming can split populations of the same species into morphs, they may differ in shape within a single ‘fragmented’ river (Acar & Kaymak, 2023). Overall, those studies and ours show consistency in the results of populations from lentic sites with less elongated heads and deeper bodies, although they evaluated changes along a ‘fragmented’ river and we did so through historical collections pre‐ and post‐damming. This change in fish shape is recorded in slow and homogeneous sites, either induced by dams or less drastic human alterations such as channelling (Petrosino et al., 2023). Phenotypic plasticity has an effect on within‐lineage changes driven by distinct environmental pressures (Acar & Kaymak, 2023; Petrosino et al., 2023). Alternatively, damming might also lead to ‘homogenization’ (or hybridization) between species once isolated by natural barriers such as waterfalls, which turned into inundated areas (Ito et al., 2023). Although *S*. *naso* is found in a semi‐enclosed area (i.e. Iguape Bay to the lower Paraguaçu River channel), it is restricted to estuarine zones mostly due to its biology. Most cases of body shape variation are recorded in rivers that have been turned into reservoirs, here, the dam has affected the estuarine part of the Paraguaçu River channel by reducing its natural freshwater flow into Iguape Bay and then into Todos os Santos Bay. We must acknowledge that old‐specimens preservation may have biased our data to some extent (see discussion in Maayan et al., 2022), although a proxy for body depth (i.e. taken from pectoral‐fin base) and head elongation still supports this morphological change. Interestingly, morphological variation recorded by Acar and Kaymak (2023) and Gilbert et al. (2020) was in a timeframe like ours (i.e. 20–35 years post‐dam construction). This temporal pattern has been explored by Cureton and Broughton (2014), who show a trend in the direction of change (e.g. towards deeper bodies) and argue that directional selection may operate in the first 10–30 years, after which stabilizing selection maintains this new shape. This may provide further support for the aforementioned findings and the likelihood of some degree of morphological change on an ecological time scale (Carroll et al., 2007). As we did not have a thorough temporal sampling, further standardized analysis could look at how these environmental pressures affect shape, when this change occurs, and whether it depends on the life history of the fish. In addition, such standardized analysis could avoid the bias associated with the preservation of old specimens.

Herein, the type locality is placed around 15 km downstream of its original assumed place. Although this update may seem small, there is a critical need for more accurate locations to avoid taxonomic and biological confusion, and to improve conservations assessments (Aguiar et al., 2021; Medeiros et al., 2022). In the case of *S*. *naso*, this update merges the biological aspects of this species with the knowledge of human‐made changes impacting the river channel and estuarine zone. Although nowadays specimens of *S*. *naso* can be recorded at least further 5 km upstream, and likely even closer to the dam, this does not represent the conditions similar to its discovery and description by Jordan and Eigenmann (1889). This accurate assessment of the type locality of *Stellifer naso* using historical grounds yields proper geographic placement and spelling corrections, which should give a base for subsequent studies of conservation, biogeography and taxonomy.

## AUTHOR CONTRIBUTIONS

**Jonas Andrade‐Santos**: conceptualization, data curation, formal analysis, funding acquisition, investigation, methodology, project administration, resources, software, validation, visualization, writing – original draft, writing – review & editing. **Marcelo R. Britto**: conceptualization, funding acquisition, investigation, project administration, resources, supervision, validation, visualization, writing – review & editing.

## FUNDING INFORMATION

The Article Processing Charge for the publication of this research was funded by the Coordenação de Aperfeiçoamento de Pessoal de Nível Superior – Brasil (CAPES) (ROR identifier: 00x0ma614).

## Footnotes

^1^

mczbase.mcz.harvard.edu/guid/MCZ:Ich:4583; mczbase.mcz.harvard.edu/guid/MCZ:Ich:10808.
